# Seasonally adjusted laboratory reference intervals to improve the performance of machine learning models for classification of cardiovascular diseases

**DOI:** 10.1186/s12911-024-02467-6

**Published:** 2024-03-04

**Authors:** Victorine P. Muse, Davide Placido, Amalie D. Haue, Søren Brunak

**Affiliations:** 1 Novo Nordisk Foundation Center for Protein Research, Faculty of Health and Medical Sciences, University of Copenhagen, 2200 Copenhagen, Denmark; 2 Copenhagen University Hospital, Rigshospitalet, Blegdamsvej 9, 2200 Copenhagen, Denmark

**Keywords:** Diagnostics, Laboratory Values, Seasonality, Machine Learning, Cardiovascular Disease, Digital Health, Electronic Health Records

## Abstract

**Background:**

Variation in laboratory healthcare data due to seasonal changes is a widely accepted phenomenon. Seasonal variation is generally not systematically accounted for in healthcare settings. This study applies a newly developed adjustment method for seasonal variation to analyze the effect seasonality has on machine learning model classification of diagnoses.

**Methods:**

Machine learning methods were trained and tested on ~ 22 million unique records from ~ 575,000 unique patients admitted to Danish hospitals. Four machine learning models (adaBoost, decision tree, neural net, and random forest) classifying 35 diseases of the circulatory system (ICD-10 diagnosis codes, chapter IX) were run before and after seasonal adjustment of 23 laboratory reference intervals (RIs). The effect of the adjustment was benchmarked via its contribution to machine learning models trained using hyperparameter optimization and assessed quantitatively using performance metrics (AUROC and AUPRC).

**Results:**

Seasonally adjusted RIs significantly improved cardiovascular disease classification in 24 of the 35 tested cases when using neural net models. Features with the highest average feature importance (via SHAP explainability) across all disease models were sex, C- reactive protein, and estimated glomerular filtration. Classification of diseases of the vessels, such as thrombotic diseases and other atherosclerotic diseases consistently improved after seasonal adjustment.

**Conclusions:**

As data volumes increase and data-driven methods are becoming more advanced, it is essential to improve data quality at the pre-processing level. This study presents a method that makes it feasible to introduce seasonally adjusted RIs into the clinical research space in any disease domain. Seasonally adjusted RIs generally improve diagnoses classification and thus, ought to be considered and adjusted for in clinical decision support methods.

**Supplementary Information:**

The online version contains supplementary material available at 10.1186/s12911-024-02467-6.

## Background

Machine learning (ML) models for use in digital medicine have been continuously advancing for years with mounting potential to come as technology and data collection procedures are enhanced in parallel [1, 2]. Increasing electronic health record (EHR) data availability and capture represent a vast opportunity to improve health care related ML models at the pre-processing level for the data they receive. This study investigates if ML-based classification of cardiovascular disease (CVD) diagnoses improves following seasonal adjustment of laboratory data reference intervals (RIs).

Seasonal patterns within laboratory values as well as disease occurrences such as CVD is a widely known and accepted phenomenon [3]. However, this knowledge regarding seasonal variation is largely unused in clinical settings despite the fact that many experts acknowledge seasonality’s profound influence on over- and under-diagnoses [4–6]. These natural fluctuations may result in prolonged diagnostic periods and unnecessary medication use by patients suffering from a misclassification of their respective test results. Additionally, current CVD models and risk scores, although well established and numerous, will notably over or under-estimate risk when tested on populations other than those they were developed on, creating a need for improved pre-processing tools to be created for population-specific model development [7, 8].

Laboratory test results are routinely classified by standard reference intervals (RI) as normal or abnormal (95% confidence interval band), as defined by national health authorities, for example the International Federation for Clinical Chemistry [4, 9, 10]. In standard care they generally do not accommodate for variation in laboratory data attributable to seasonal fluctuations; a mainstream example being vitamin D which is driven to fluctuate seasonally by sun exposure [11–14]. Other widely known seasonally varying laboratory tests include thyroid stimulating hormone (TSH) and vitamin B12, driven by temperature changes and seasonal diet changes respectively [15–19]. White blood cells and other immune markers also display seasonality shifts due to changes in allergens in the environment [5, 13, 20].

CVDs are classified as an epidemic given their ranking as the leading cause of death worldwide with 17.9 million deaths per year [21]. International calls to action to address this public health crisis reference the vast opportunities available to researchers today that never existed before: a major one being the cumulative knowledge and data available [22, 23]. While seasonal variations in incidence of CVDs are widely known, the underlying mechanisms remain unknown but are broadly described as a complex interplay between human physiology with its environment [3]. Accordingly, it is well-known that responses to the environment, such as temperature fluctuations, are reflected in the blood composition and components [24]. However, this knowledge has not yet been translated into clinical practice, and has never been studied in a large-scale, data driven study.

In response, this study specifically focuses on optimization of the ML performance for classification of diagnoses in the International Classification of Diseases 10th revision (ICD-10) code chapter IX: Diseases of the Circulatory system, because the foundational method of this study, Muse et al., identified strong correlations between ICD-10 classifications and seasonally adjusted laboratory values [3, 6, 25–27]. We assessed if four different ML models trained to classify hospital admissions according to the CVD improved their performance after the modification to seasonally adjusted RIs. Input features were laboratory test results taken within 24 h of admission and patient sex. Results from this study ultimately show the long term and comprehensive benefits of proper lab data cleaning and pre-processing procedures for use in future large-scale ML clinical data-based projects.

## Methods

### Data overview

Population-wide laboratory in-patient data from two Danish health regions during years 2012 to 2015 (inclusive) were included in this analysis. The input dataset was processed and cleaned systematically by standardizing test names to English, normalizing units, and removing numerical typos such as extra commas or spaces, as introduced in Muse et al. [27] Only data from patients aged >  = 20 were included in the study. The RIs for these laboratory data were then seasonally adjusted (described in detail below), and only tests with a significant seasonality fit were included as independent variables in the ML models.

**Fig. 1:**
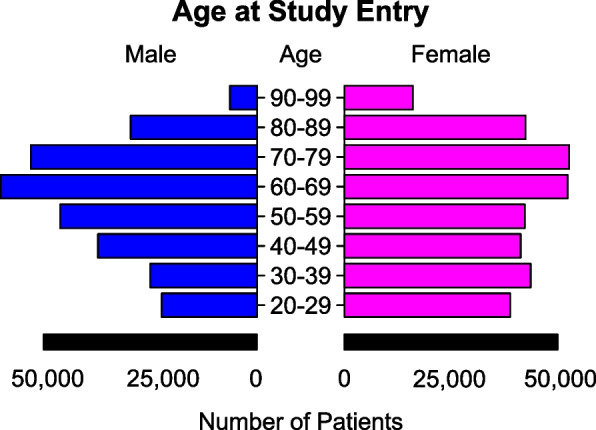
Sex and age distributions for all included patients at time of entry for the study

Patient IDs present in the laboratory dataset were then identified in the Danish National Patient Registry (DNPR). Denmark has had continuous record keeping using a person identification system since 1968 and it is therefore possible to accurately link patient records over time [28, 29]. Admissions linked to the laboratory data were used to build training and validation data for specific ML prediction tasks. To link records from admissions with hospital transfers, hospital encounters less than 24 h apart were pieced together. For each admission we collected all the corresponding laboratory values within 24 h after the timestamp of admission. In cases of repeated test measurements for the same patient within 24 h of admission, only the most abnormal test result was included: i.e., + 1 or -1, given that the patient would present with symptoms that could be improved by physicians within the 24-h window. Figure 1 details the final cohort’s sex and age distributions and Table 1 details total record availability.

**Table 1:** Data availability overview of unique patient, laboratory test, and hospital encounter data

	**Female**	**Total**
**Unique Patients Available**		
	303, 638 (54%)	561,368 (100%)
**Total Records Available**		
Laboratory Tests	8,670,978 (51%)	17,137,742 (100%)
Hospital Encounters	731,820 (51%)	1,421,926 (100%)

### Seasonal adjustment of laboratory data

Data processing and stratum definition requirements were introduced to ensure high quality data retention. An individual stratum is defined by unique combinations of laboratory test, unit, lab ID, sex, and age group. Unique laboratory tests are defined by distinct combinations of a test name, and source specimen (i.e., plasma, urine, etc.). Examples of different unique tests are Albumin – P and Albumin – U, albumin from plasma and urine respectively. Age groups were defined as 10-year periods. For example, one age group included 20-year-olds to 30-year-olds.

Methods and data processing relating to calculating sex and age specific sinusoidal fits were conducted in a manner similar to other studies [4, 27]. Data for each unique above defined stratum from the four inclusive years (2012–2015) was normalized to 0 and correspondingly fit to Eq. 1 as sinusoidal models are good at capturing temporal data with one peak and one trough. To focus on natural seasonal variation, only data in which the patient survived more than 28 days was included when adjusting for seasonality trends, as to not bias results towards critically ill patient profiles.

1 $$y={\beta }_{0}+{\beta }_{1}*({\text{cos}}\left(2*\pi *\frac{week-\theta }{52}\right))$$

Parameter fitting was conducted using a Non-linear Least Squares (NLS) algorithm to Eq. 1using R software (version 4.0.0) [27]. Laboratory tests stratified by age and sex group were classified as having a significant seasonality shift if their respective parameters, defined by ${\beta }_{0}$, ${\beta }_{1}$, and $\theta$ fit to Eq. 1 with p value < 0.05, FDR corrected by parameter, as reported by the NLS R software package. Parameters “height” (${\beta }_{0}$) and “amplitude” (${\beta }_{1}$) were bounded to float between -1, and 1, while “offset” ($\theta$) was bounded between 0 and 52 using the “port” algorithm [27, 30]. A new RI was calculated by applying this calculated seasonality fit to the reported standard RI. This step was accomplished by taking the fitted wave function for each specific test, age, and sex group and inputting the corresponding $\theta$ value to calculate the new RI for each record. For laboratory tests that did not display significant seasonality shifts we maintained the standard RIs as defined by health authorities, and accordingly these tests are not assessed in this study. The 23 tests that were classified as having significant seasonality changes and measured in at least 20% of admissions within 24 h of the given hospital encounter were included as input features for the ML models. The 23 tests are listed in supplementary Table 1. We acknowledge that inclusion of all other data would likely further improve the model, but that is not the main goal of this study as we focus on the effect of RI adjustment. Each of the included laboratory test measurements were then assigned two features, one if the test was abnormal with the original, un-adjusted, RI (termed “version 1”), and one if the test was considered abnormal after the RI was seasonally adjusted (termed “version 2”).

### Selection of chapter IX level 3 ICD-10 codes

Once the laboratory dataset had been defined and adjusted RIs had been introduced, patients were identified in the in-patient admission data from the DNPR. ICD-10 chapter IX codes were collapsed to level 3 codes (e.g. I21.2 was converted to I21) to better assess high level disease trends in the ML model. Accordingly, only ICD-10 codes with at least 1,000 unique patients, were included. This step resulted in 35 unique level 3 ICD-10 codes, which are reported and annotated in supplementary Table 1. Incidence rates of each diagnosis in the studied population is reported in supplementary Table 2.

### Machine learning model features

The outcomes were defined as the set of primary and secondary ICD-10 codes registered at each admission. A binary classifier was trained separately for all the unique 35 level 3 ICD-10 codes that were selected via stratum requirements previously described. Each model was trained on sex and the set of 23 laboratory tests, where + 1 encoded lab-values above the reference interval, -1 lab-values below the interval, and 0 a lab-value within the interval. The reference interval was defined depending on the criteria version 1 vs version 2, as previously defined.

**Fig. 2:**
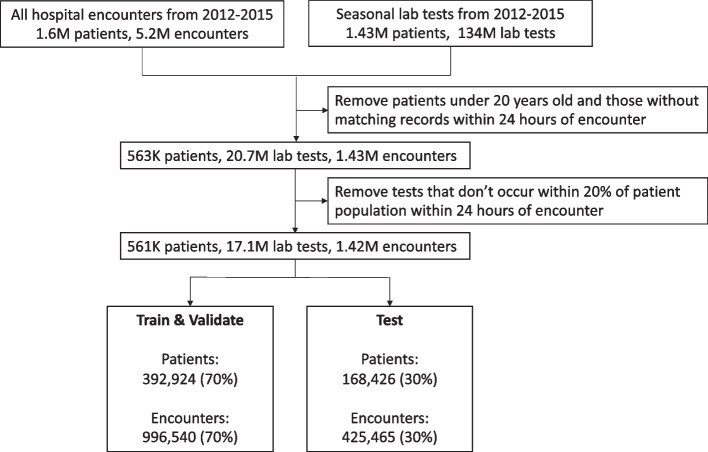
Attrition diagram detailing data preprocessing steps. The final data set is also summarized in Fig. 1

Missing values were imputed with constant value 0 (representing tests within the reference interval), then input values were standardized by subtracting the mean and scaling to unit variance. This is the standard practice for laboratory test imputing in other studies due to physician testing protocols in Denmark [27, 31, 32]. The dataset was randomly divided into a development and test set (70% and 30% of the original data, respectively) using the unique patient ID, detailed in Fig. 2. In this way admissions of the same patients were labelled with the same split. The development set was used in a random search optimization, where a maximum of 40 combinations were sampled from the hyperparameter distribution and fitted using fivefold cross-validation. In order to adjust for diseases with low prevalence during training, we resampled each batch with balanced numbers of cases and controls.

We developed and tested four ML models: adaBoost, decision tree, neural net, and random forest [33]. Supplementary Table 3 shows which hyperparameter distributions were used for each model. For each classifier, the best model selected using the hyperparameter configuration with highest F1 score in the development set was used to calculate the final performances on the test set. Given the different incidence of the outcomes, classification performances were assessed using both area under precision recall (AUPRC) and area under receiver operator characteristic (AUROC), reported in detail in supplementary Table 4. Confidence intervals were constructed using 1,000 bootstrap samples and differences in AUROC and AUPRC were calculated by pairing boot samples (the same admissions were included in the same boot by setting the same random seed for both versions) [34]. These distributions of differences per model were then assessed for significant changes using the accelerated bootstrap method (10,000 boots, bcaboot package in R), adjusting for potential bias in the samples [35]. Reported median net changes ( ±) between versions 1 and 2 were considered significant if the reported 95% CI from this method did not include 0.

For each neural net ICD-10 model we calculated the features’ contribution approximating the SHapley Additive exPlanation (SHAP) values by iterating through permutations of the input. Among the ML models, the neural net was chosen to be investigated in further detail for this study because the total net gain for the seasonal model across all disease codes was highest (14%). The one-way ANOVA test was used to compare the distributions of the SHAP values associated to the different values of a given laboratory test. Multiple testing correction was performed using the Bonferroni method, after which features were deemed significant if the p value was still below 0.05. Mean absolute SHAP values for all neural net models are reported in supplementary Table 5. Analyses were performed using Python v3.9.13 (package scikit learn v1.0.2 for model development and validation) [33, 29].

## Results

### Cohort overview

Processing requirements, as described in Methods, narrowed the available data set to 17,137,742 unique records, 1,421,926 admissions, 561,368 unique patients, 23 unique laboratory tests, and 35 level 3 ICD-10 codes for testing in the proposed ML models (detailed in Figs. 1 and 2, Table 1, and supplementary Table 1). This reduction in data was needed to specifically capture the effect seasonal adjustment has on ML model prediction, as a test case for more disease specific models that would still use the entirety of the laboratory dataset. Chapter IX ICD-10 level 3 codes were only considered if they were assigned during an admission where at least one of the included laboratory tests was measured.

As seen in Fig. 1, sexes are generally equally represented apart from ages 20–40 where there are proportionally more women than men in the dataset. This trend is expected as the population represents women being admitted for pregnancy related events. For ages 50–79, males and females were equally represented, whereas patients > 80 years were dominated by females consistent with the fact that life expectancy for females is longer than that of males (Fig. 1).

### ML performance metric results

**Fig. 3:**
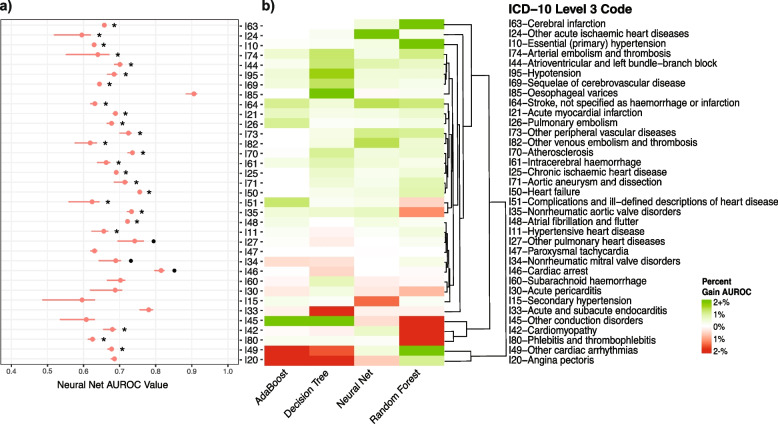
**(a)** AUROC performance metric values for the version 2 neural net model. Dots indicate the median AUROC values and associated lines show the corresponding 95% CIs. The symbol (*) indicates that the version 2 model performed significantly better than the version 1 model (with respect to accelerated bootstrap gains 95% CIs) and the symbol (•) indicates that the models performed the same statistically; no symbol indicates the model performed significantly worse (with respect to accelerated bootstrap gains 95% CIs). **(b)** Heatmap of net AUROC gains (version 2—version 1) across all available ICD-10 chapter IX codes for the four ML models assessed in this study. Clustering along the Y-axis was performed using hclust algorithm in R. The scale was bounded between + 2% and -2% to enable easier viewing, although net gains can be higher or lower, as listed in supplementary Table 4. Non-significant gains/ losses (95% CI accelerated bootstrap gains didn’t include 0) were changed to “0” before performing the clustering and are therefore represented as white. All corresponding values are also included in supplementary Table 4

Figure 3a displays the results of the neural net AUROC performances metric with seasonal adjustment (supplementary Fig. 1 shows the results for the non-seasonally adjusted version). The figure demonstrates that seasonally adjusted RI models (version 2) improved ML-based CVD classification. Overall, 92 of the 140 studied experiments were classified with significantly better accuracy (based on AUROC improvements) after seasonal adjustment across the four models; for neural net models specifically, 24 of 35 meet this threshold. While some of these gains at the disease level can be quite small (< 0.1%), the total net gain across the neural net models resulted in a + 14% improvement, followed by random forest: + 7.8%, decision tree: + 1.2%, and adaBoost: -5.9%.

Figure 3b compares the net AUROC gains across the four ML models and the models generally captured similar trends. For 18 of the codes, all four models had the same or improved AUROC performance, whereas there are disagreements across the four models for 14 codes. Stroke, not specified as hemorrhage or infarction (I64), essential (primary) hypertension (I10), atrioventricular and left bundle-branch block (I44), pulmonary embolism (I26), heart failure (I50), and atrial fibrillation and flutter (I48) are examples of diagnoses with consistent positive net gains across the four models. In contrast, acute and subacute endocarditis (I33) was the only diagnoses with a consistent negative net gain across the four different models. Random forest demonstrated the largest variation in net AUROC gains ranging from – 8% to + 9%.

Classification of diseases of the vessels, such as arterial embolism and thrombosis (I74), stroke, not specified as hemorrhage or infarction (I64), other peripheral vascular diseases (I73), atherosclerosis (I70), acute myocardial infarction (I21), and aortic aneurysm and dissection (I71) consistently improved for seasonally adjusted RIs (Fig. 3b). Interestingly, the classification of essential hypertension (I10) improved following seasonal adjustment whereas the classification of secondary hypertension (I15), generally did not (Fig. 3b). This is consistent with the fact that essential hypertension (I10) is a major lifestyle disease and thus would be expected to co-vary with seasonal trends, while secondary hypertension is caused by other diseases, and therefore not likely to be seasonally driven.

### Feature analyses by SHAP

**Fig. 4:**
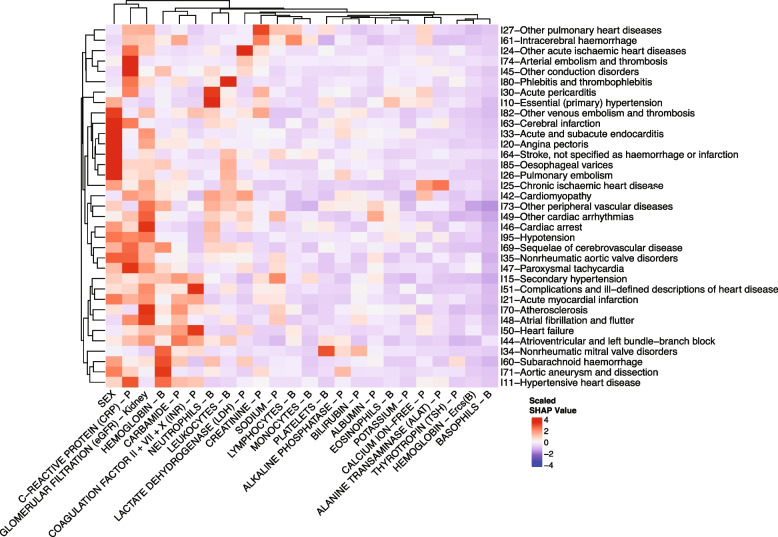
Heatmap of scaled SHAP values by ICD-10 chapter IX code for the version 2 neural net model. The value of each cell is calculated as the mean of the absolute SHAP values across all admissions. Values are scaled by ICD-10 code and can therefore be compared empirically as a ranking across ICD-10 codes where red indicates highest feature importance to the model, and blue indicates lowest feature importance to model. All absolute mean SHAP values for versions 1 and 2 neural nets are reported in supplementary Table 5

Since total net gains for were highest for the neural net across all disease codes, we further characterized the model results using SHAP values by plotting the mean of the absolute SHAP values for the 23 laboratory tests and sex features against the 35 diagnoses in Fig. 4. The feature category “sex” serves as a positive control since the diagnoses with high absolute mean SHAP values have known differences in prevalence among sexes. Some key examples include angina pectoris (I20), cerebral infarction (I63), and pulmonary embolism (I26). Interestingly, the trends for C-reactive protein (CRP) and leukocytes (both markers of infection and inflammation) did not display similar trend: the feature contribution of CRP was generally high and the feature contribution for leukocytes was generally low (Fig. 4). After sex, CRP, eGFR and hemoglobin demonstrated the net greatest model attribution on the left. Interestingly, these three tests are also among the most used lab tests. And generally, markers of inflammation had a high model attribution. The signals for electrolytes were less consistent, as SHAP values for potassium were generally negative and equally positive and negative for sodium. Moreover, SHAP values for ALAT and free calcium were considerably high in classification of chronic ischemic heart disease (I25) and hypertensive disease (I11) whereas the mean absolute SHAP value of these two input features were low for the remaining 33 diagnoses.

**Fig. 5:**
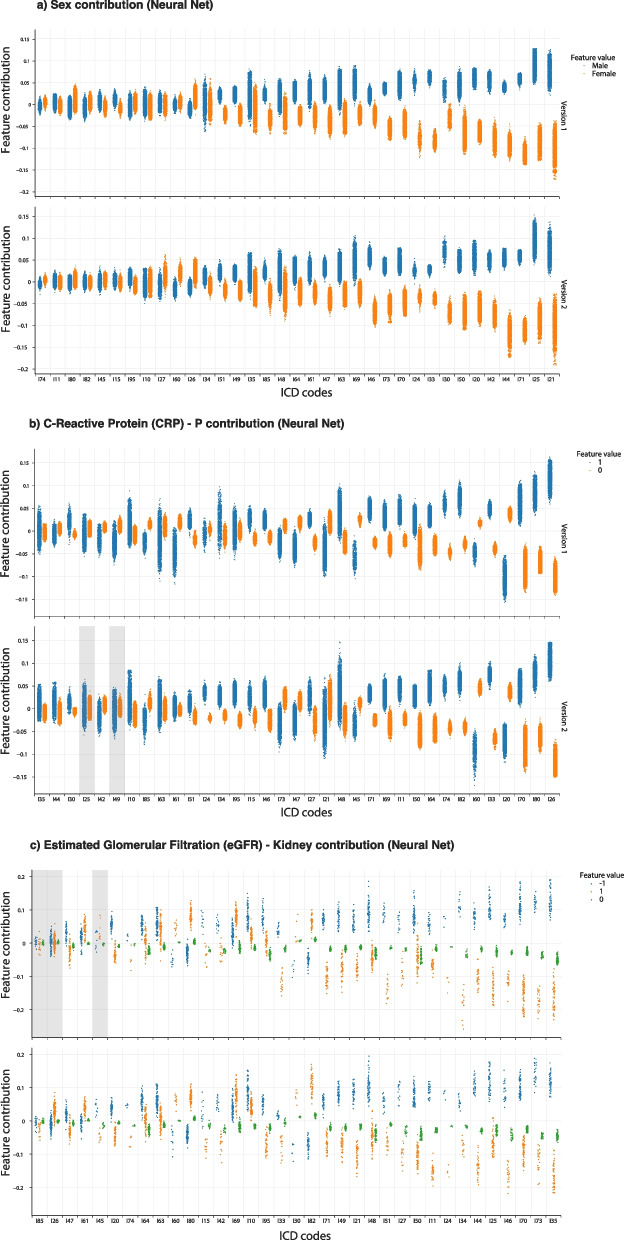
**(a-c)** Plot of SHAP values as estimate of feature contribution by ICD-10 chapter IX code for the versions 1 and version 2 Neural Net model for the three most important features across all of the ICD-10 codes. ICD-10 codes are sorted by absolute mean SHAP value, i.e., from left to right the contribution of the laboratory test increases. Shaded bands indicate there was no significant contribution difference for the given level 3 ICD-10 code/ model version (p value < 0.05, Bonferroni corrected). Supplementary Table 5 reports all mean absolute SHAP values for Neural Net version 1 and 2 models

Figure 5 displays the feature contribution (quantified by SHAP values) of the four different features with the highest overall importance for each of the 35 ICD-10 codes for version 1 (upper panel) and version 2 (lower panel) highlighted in Fig. 4. Figure 5a served as a positive control case as the feature inputs never changed (i.e., sex input values did not change between versions 1 and 2). While some contributions increased or decreased, the directionality of the contribution was the same for all ICD-10 codes, as expected. Overall, similar trends were observed when comparing the SHAP values for versions 1 and version 2 indicating that feature contributions were independent of seasonal adjustment, with notable exceptions. There were only few cases with no statistically significant difference between SHAP values for different values of the input feature (indicated by the gray bands). Generally, values below reference range for eGFR had positive feature contributions (Fig. 5c). Values above reference range for CRP had high feature importance while the opposite was generally seen for values within reference range (Fig. 5b). For several diagnoses, however, there were differing trends. For example, for pulmonary embolism (I26) SHAP values for eGFR had no significant feature contribution in version 1, whereas there were significant differences in SHAP contributions for eGFR values in version 2.

## Discussion

In this study, we present the first application of a method for studying the impact of seasonal variation of laboratory test results on CVD diagnoses classifier models. We developed four ML models based on 1,421,926 hospital encounters from 561,368 unique patients and found that the AUROC metrics for diagnostic classification significantly improved for 24 of 35 CVD diagnostic codes for the model with the best overall performance. While this study focuses on diseases of the circulatory system, there are endless applications to other disease domains related to well-known seasonally effected laboratory values such as vitamin D, vitamin B12, TSH, and a multitude of immune parameters in addition to areas where these relations are yet to be discovered [11, 16, 19, 20].

Overall, the four ML models displayed similar results although there was some variation. Heart failure was one of the many diagnoses where the classification performance generally improved after seasonally corrected RIs. Thus, this study adds a possible mechanistic insight into the fact that there is a “winter peak” in the occurrence of heart failure [36]. However, net gains for acute and subacute endocarditis (I33) were consistently less than zero. This finding is consistent with previous studies that report a lack of seasonality occurrence in endocarditis [37]. This further highlights the complexity of seasonal adjustment in disease classification modeling and supports the argument that development of new methods within this domain are necessary, as has been similarly called for in other studies involving biological modeling [38].

We chose to develop four distinct ML models, to assess the robustness of the value of data integration based on such models. To this end, we argue that the overall results with model improvement across the board in over half of the cases underline the potential of acknowledging seasonal variation in clinical laboratory data. For example, we found that markers of inflammation generally had high model attribution, whereas electrolytes generally had lower model attribution (Fig. 4). In fact, we argue that our observations explain some of the seasonal variation that have been cemented in observational studies and add evidence to existing theories regarding seasonality in disease prevalence [36, 39, 32, 34]. Yet, we are aware that the exact mechanisms are far from understood and that other factors not reflected by the laboratory data ought to be considered.

The results highlighted in Fig. 5a proved to be an important positive control result for this study because the direction of contribution stayed consistent for all disease codes across versions 1 and 2. More importantly, we see that in most cases being male increases feature importance which is consistent with the well-known fact that males are at higher risk for developing CVD. Figure 4 solidifies this fact since sex is identified as one of the higher-ranking features for most ICD-10 codes, and the highest being I21: acute myocardial infarction.

Taken together, we have explored the potential ML performance gains available to researchers with improved data pre-processing steps, specifically non-pathological seasonality shifts in laboratory test results caused by natural weather and dietary shifts throughout the year. While other studies have confirmed the diagnostic error potential that exists due to seasonality induced variation, none have studied these seasonal shifts comprehensively. Our results serve as a proof-of-principle that seasonal shifts in laboratory data do in fact impact diagnostics and thus ought to be considered – at least in the Danish setting. We argue that these findings are of value because the clinical manifestation of these diseases are prone to correlate with external factors, usually characterized by an inherent periodicity.

Future studies using an adjustment method such as the one presented here, are expected to have even better performance gains since the model can be fit to disease specific diagnostic windows and exploit other non-linear correlations. This study made use of a more generic 24-h window to investigate trends at a high level. Some diseases can take days, weeks, or months to diagnose in which case their corresponding performance metric gains would not be seen in this study. Future models can also investigate disease trajectories, prognostics or even mortality risk assessment, instead of the simpler disease classification ML model presented here. In addition, this study only highlighted performance gains for seasonally adjusted data, and thus future studies will use all available data (i.e., seasonally adjusted RI tests as well as standard RI tests) to optimize ML performance.

## Conclusions

In sum, this study succeeds in demonstrating that ML-based classification of CVDs models could benefit from seasonally-relevant data pre-processing steps in future EHR-based studies. While physicians may better understand the nuances and seasonal variation in their patient's laboratory results, current computational based models rarely adjust for such naturally occurring trends and drift. With growing access to large patient cohort data, pre-processing tools such as the one presented in the study could be a key factor to the next generation of diagnostic classification models both for CVD and many other diseases.

### Supplementary Information

**Supplementary Material 1. **

## Data Availability

The data that support the findings of this study are not publicly available as they contain person sensitive information. To obtain access to data, the study needs to be approved by the Danish authorities including the Danish Data Protection Agency (www.datatilsynet.dk) All studies should be conducted in compliance with The Danish Act on Processing of Personal Data and all other applicable laws and regulations. This paper does not report original code.

## Abbreviations

CRP: C-Reactive Protein
CVD: Cardiovascular Disease
DNPR: Danish National Patient Registry
EHR: Electronic Health Record
ICD: International Statistical Classification of Diseases
IFCC: International Federation of Clinical Chemistry;
ML: Machine Learning
NLS: Non-linear least-squares
RI: Reference Interval
TSH: Thyroid Stimulating Hormone
