# Supplementary material for: Seasonally adjusted laboratory reference intervals to improve the performance of machine learning models for classification of cardiovascular diseases
Source: BMC Med Inform Decis Mak. 2024 Mar 4;24:62. doi: 10.1186/s12911-024-02467-6 (PMC10910795; doi:10.1186/s12911-024-02467-6)
Supplement: Supplementary file 1 — Supplementary Material 1. [file 12911_2024_2467_MOESM1_ESM.docx]

**Seasonally modified laboratory reference intervals to improve the performance of machine learning models for classification of cardiovascular diseases**

Victorine Muse^*,1^, Davide Placido^*,1^, Amalie Dahl Haue^1,2^, and Søren Brunak^†,1,2^

^1^Novo Nordisk Foundation Center for Protein Research, Faculty of Health and Medical Sciences, University of Copenhagen, DK-2200 Copenhagen, Denmark

^2^Copenhagen University Hospital, Rigshospitalet, Blegdamsvej 9, DK-2200 Copenhagen, Denmark.

^†^Email: [soren.brunak@cpr.ku.dk](mailto:soren.brunak@cpr.ku.dk)

**Supplementary Table 1:** Laboratory Test and ICD-10 code study inclusion overview. Laboratory tests are only included if 20% of all admissions had a value taken within 24 hours of the hospital encounter. A minimum of 1,000 unique patients are required to receive the corresponding level 3 ICD-10 code diagnoses to be retained in the study.

| **Laboratory tests assessed** | **Level 3 ICD-10 codes assessed** |
| --- | --- |
| ALANINE TRANSAMINASE (ALAT) – P, ALBUMIN – P, ALKALINE PHOSPHATASE – P, BASOPHILS – B, BILIRUBIN – P, C-REACTIVE PROTEIN (CRP) – P, CALCIUM ION-FREE – P, CARBAMIDE – P, FACTOR II + VII + X (INR) – P, CREATININE – P, EOSINOPHILS – B, GLOMERULAR FILTRATION (EGFR) – Kidney, HEMOGLOBIN - B, HEMOGLOBIN - Ercs(B), LACTATE DEHYDROGENASE (LDH) – P, LEUKOCYTES – B, LYMPHOCYTES – B, MONOCYTES – B, NEUTROPHILS – B, PLATELETS – B, POTASSIUM – P, SODIUM – P, THYROTROPIN (TSH) - P. | I10: Essential (primary) hypertension; I11: Hypertensive heart disease; I15: Secondary hypertension; I20: Angina pectoris; I21: Acute myocardial infarction; I24: Other acute ischemic heart diseases; I25: Chronic ischemic heart disease; I26: Pulmonary embolism; I27: Other pulmonary heart diseases; I30: Acute pericarditis; I33: Acute and subacute endocarditis; I34: Nonrheumatic mitral valve disorders; I35: Nonrheumatic aortic valve disorders; I42: Cardiomyopathy; I44: Atrioventricular and left bundle-branch block; I45: Other conduction disorders; I46: Cardiac arrest; I47: Paroxysmal tachycardia; I48: Atrial fibrillation and flutter; I49: Other cardiac arrhythmias; I50: Heart failure; I51: Complications and ill-defined descriptions of heart disease; I60: Subarachnoid hemorrhage; I61: Intracerebral hemorrhage; I63: Cerebral infarction; I64: Stroke, not specified as hemorrhage or infarction; I69: Sequelae of cerebrovascular disease; I70: Atherosclerosis; I71: Aortic aneurysm and dissection; I73: Other peripheral vascular diseases; I74: Arterial embolism and thrombosis; I80: Phlebitis and thrombophlebitis; I82: Other venous embolism and thrombosis; I85: Esophageal varices; I95: Hypotension; |

**Supplementary Table 2:** Disease occurrence rate by level 3 ICD-10 code. The cohort in this study includes 561,368 total unique patients (54% female) and 1,421,926 total unique hospital encounters (51% female) as summarized in the main paper in **figure 1a**. These numbers are used to calculate reported percentages below. Total disease occurrence for the ICD-10 chapter IX codes assessed in this study is 191,398 patients, 389,470 encounters, accounting for 34.1% percent and 27.4% percent of records respectively.

| **ICD-10 Code** | **Level 3 ICD-10 Name** | **Total ICD-10 occurrence by patient (% total)** | **Female ICD-10 occurrence by patient (% positive patients)** | **Total ICD-10 occurrence by hospital encounter (%total)** | **Female ICD-10 occurrence by hospital encounter (% positive encounters)** |
| --- | --- | --- | --- | --- | --- |
| **I10** | **Essential (primary) hypertension** | 87831 (15.65%) | 46048 (52%) | 129565 (9.11%) | 67812 (52%) |
| **I11** | **Hypertensive heart disease** | 2966 (0.53%) | 1420 (48%) | 3317 (0.23%) | 1602 (48%) |
| **I15** | **Secondary hypertension** | 1638 (0.29%) | 849 (52%) | 2008 (0.14%) | 969 (48%) |
| **I20** | **Angina pectoris** | 13664 (2.43%) | 5507 (40%) | 18065 (1.27%) | 7041 (39%) |
| **I21** | **Acute myocardial infarction** | 17014 (3.03%) | 6123 (36%) | 25527 (1.8%) | 8748 (34%) |
| **I24** | **Other acute ischaemic heart diseases** | 1188 (0.21%) | 465 (39%) | 1285 (0.09%) | 498 (39%) |
| **I25** | **Chronic ischaemic heart disease** | 30813 (5.49%) | 10795 (35%) | 51570 (3.63%) | 17161 (33%) |
| **I26** | **Pulmonary embolism** | 7153 (1.27%) | 3681 (51%) | 9589 (0.67%) | 5000 (52%) |
| **I27** | **Other pulmonary heart diseases** | 1582 (0.28%) | 846 (53%) | 2735 (0.19%) | 1445 (53%) |
| **I30** | **Acute pericarditis** | 1300 (0.23%) | 361 (28%) | 1691 (0.12%) | 479 (28%) |
| **I33** | **Acute and subacute endocarditis** | 1006 (0.18%) | 311 (31%) | 2728 (0.19%) | 759 (28%) |
| **I34** | **Nonrheumatic mitral valve disorders** | 2096 (0.37%) | 1014 (48%) | 3314 (0.23%) | 1556 (47%) |
| **I35** | **Nonrheumatic aortic valve disorders** | 7506 (1.34%) | 3484 (46%) | 13316 (0.94%) | 6051 (45%) |
| **I42** | **Cardiomyopathy** | 2670 (0.48%) | 1004 (38%) | 3929 (0.28%) | 1482 (38%) |
| **I44** | **Atrioventricular and left bundle-branch block** | 4645 (0.83%) | 1715 (37%) | 6027 (0.42%) | 2207 (37%) |
| **I45** | **Other conduction disorders** | 1779 (0.32%) | 783 (44%) | 2079 (0.15%) | 912 (44%) |
| **I46** | **Cardiac arrest** | 3693 (0.66%) | 1289 (35%) | 4502 (0.32%) | 1526 (34%) |
| **I47** | **Paroxysmal tachycardia** | 6409 (1.14%) | 3196 (50%) | 8761 (0.62%) | 4135 (47%) |
| **I48** | **Atrial fibrillation and flutter** | 49633 (8.84%) | 22783 (46%) | 97805 (6.88%) | 44302 (45%) |
| **I49** | **Other cardiac arrhythmias** | 6973 (1.24%) | 3347 (48%) | 8701 (0.61%) | 4223 (49%) |
| **I50** | **Heart failure** | 26036 (4.64%) | 10522 (40%) | 47850 (3.37%) | 17619 (37%) |
| **I51** | **Complications and ill-defined descriptions of heart disease** | 1660 (0.3%) | 758 (46%) | 1993 (0.14%) | 884 (44%) |
| **I60** | **Subarachnoid haemorrhage** | 1528 (0.27%) | 841 (55%) | 2242 (0.16%) | 1299 (58%) |
| **I61** | **Intracerebral haemorrhage** | 3430 (0.61%) | 1614 (47%) | 4707 (0.33%) | 2154 (46%) |
| **I63** | **Cerebral infarction** | 18784 (3.35%) | 8817 (47%) | 22886 (1.61%) | 10608 (46%) |
| **I64** | **Stroke, not specified as haemorrhage or infarction** | 9617 (1.71%) | 4587 (48%) | 11265 (0.79%) | 5344 (47%) |
| **I69** | **Sequelae of cerebrovascular disease** | 19509 (3.48%) | 9098 (47%) | 28920 (2.03%) | 13117 (45%) |
| **I70** | **Atherosclerosis** | 5785 (1.03%) | 2395 (41%) | 8607 (0.61%) | 3469 (40%) |
| **I71** | **Aortic aneurysm and dissection** | 3485 (0.62%) | 1025 (29%) | 5665 (0.4%) | 1609 (28%) |
| **I73** | **Other peripheral vascular diseases** | 3795 (0.68%) | 1608 (42%) | 5262 (0.37%) | 2256 (43%) |
| **I74** | **Arterial embolism and thrombosis** | 1105 (0.2%) | 534 (48%) | 1344 (0.09%) | 660 (49%) |
| **I80** | **Phlebitis and thrombophlebitis** | 10164 (1.81%) | 5209 (51%) | 11966 (0.84%) | 6055 (51%) |
| **I82** | **Other venous embolism and thrombosis** | 2295 (0.41%) | 1147 (50%) | 2681 (0.19%) | 1366 (51%) |
| **I85** | **Oesophageal varices** | 1188 (0.21%) | 388 (33%) | 2436 (0.17%) | 765 (31%) |
| **I95** | **Hypotension** | 4436 (0.79%) | 2206 (50%) | 5109 (0.36%) | 2510 (49%) |

**Supplementary Table 3:** Overview of the four ML models used in this study and the corresponding hyperparameter spaces for each one.

| **Model Name** | **Hyperparameter Search** |
| --- | --- |
| Neural Network | - Hidden layer size: discrete uniform distribution 10,100 - Alpha: uniform distribution 0,1 - Batch size: discrete uniform distribution (512,1024) |
| Decision tree | - Criterion for split quality: random choice [gini, entropy] - Split: random choice [Random - best] - Max depth: discrete uniform distribution [1, 15] |
| Random forest | - Number of estimators: discrete uniform distribution [1,50] - Criterion for split quality: random choice [gini, entropy] - Maximum depth: discrete uniform distribution [1,5] |
| AdaBoost | - Number of estimators: discrete uniform distribution [1,50] |

**Supplementary Table 4:** Calculated AUROC and AUPRC for the 35 ICD-10 codes studied. All four models are shown here with median performance metric reported with the corresponding 95% confidence interval (CI) for both version 1 and version 2. The calculated net gain in performance metric is reported as version 2 – version 1. Differences in paired boot samples were assessed for significance using the accelerated bootstrap method; resulting 95% CIs that didn’t include 0 are deemed to have a significant difference between version 1 and 2.

| **ICD-10 Code** | **Model** | **Net Gain (V2-V1)** | **Metric** | **Version 1 Median score [95% CI]** | **Version 2 Median score [95% CI]** | **Accelerated Bootstrap 95% CI** |
| --- | --- | --- | --- | --- | --- | --- |
| I10 | AdaBoost | 0.0103 | AUPRC | 0.1266 [0.1254-0.1341] | 0.1369 [0.1258-0.138] | [1.03E-2,1.03E-2] |
| I10 | AdaBoost | 0.001 | AUROC | 0.5922 [0.5906-0.5929] | 0.5932 [0.5917-0.5965] | [8.49E-4,9.63E-4] |
| I10 | Decision Tree | 0.0002 | AUPRC | 0.1287 [0.1271-0.1304] | 0.1289 [0.1267-0.1304] | [1.34E-5,2.09E-4] |
| I10 | Decision Tree | 0.0017 | AUROC | 0.6022 [0.596-0.6062] | 0.6039 [0.5966-0.6066] | [1.21E-3,1.75E-3] |
| I10 | Neural Net | 1.00E-04 | AUPRC | 0.1405 [0.1391-0.1416] | 0.1406 [0.1391-0.1418] | [-6.88E-6,1.39E-4] |
| I10 | Neural Net | 0.0035 | AUROC | 0.6259 [0.6235-0.6274] | 0.6294 [0.627-0.6309] | [3.46E-3,3.65E-3] |
| I10 | Random Forest | -0.3167 | AUPRC | 0.5177 [0.1303-0.5443] | 0.201 [0.1178-0.4982] | [-2.97E-1,-1.02E-1] |
| I10 | Random Forest | 0.0427 | AUROC | 0.516 [0.5042-0.5443] | 0.5587 [0.5291-0.5732] | [3.53E-2,3.86E-2] |
| I11 | AdaBoost | 1.00E-04 | AUPRC | 0.0045 [0.0043-0.0047] | 0.0046 [0.0043-0.0047] | [2.65E-5,3.55E-5] |
| I11 | AdaBoost | 0.0015 | AUROC | 0.662 [0.6544-0.6684] | 0.6635 [0.6542-0.6711] | [1.38E-3,1.90E-3] |
| I11 | Decision Tree | 0.0061 | AUPRC | 0.0062 [0.0038-0.0124] | 0.0123 [0.0034-0.141] | [4.97E-3,7.46E-3] |
| I11 | Decision Tree | -0.0023 | AUROC | 0.6359 [0.6217-0.6475] | 0.6336 [0.6167-0.6476] | [-2.54E-3,-8.54E-4] |
| I11 | Neural Net | 0 | AUPRC | 0.0044 [0.0039-0.0048] | 0.0044 [0.0038-0.0049] | [2.71E-6,4.43E-5] |
| I11 | Neural Net | 0.0013 | AUROC | 0.6548 [0.6272-0.6663] | 0.6561 [0.6237-0.6671] | [4.05E-4,1.84E-3] |
| I11 | Random Forest | 1.00E-04 | AUPRC | 0.0044 [0.0041-0.0047] | 0.0045 [0.0043-0.0047] | [5.64E-5,8.59E-5] |
| I11 | Random Forest | 0.0041 | AUROC | 0.6612 [0.654-0.6672] | 0.6653 [0.6587-0.6709] | [3.63E-3,4.27E-3] |
| I15 | AdaBoost | 0 | AUPRC | 0.0027 [0.0025-0.003] | 0.0027 [0.0025-0.003] | [-2.42E-5,-1.50E-5] |
| I15 | AdaBoost | 0.0025 | AUROC | 0.6339 [0.6209-0.6458] | 0.6364 [0.6234-0.6485] | [2.28E-3,2.90E-3] |
| I15 | Decision Tree | -2.00E-04 | AUPRC | 0.0043 [0.0022-0.0135] | 0.0041 [0.0022-0.0124] | [-4.20E-4,-7.23E-5] |
| I15 | Decision Tree | 0.001 | AUROC | 0.6029 [0.5858-0.6191] | 0.6039 [0.587-0.6197] | [3.72E-4,1.74E-3] |
| I15 | Neural Net | -2.00E-04 | AUPRC | 0.0023 [0.0016-0.0028] | 0.0021 [0.0013-0.0027] | [-2.52E-4,-1.86E-4] |
| I15 | Neural Net | -0.017 | AUROC | 0.613 [0.5477-0.6353] | 0.596 [0.4873-0.6307] | [-1.66E-2,-1.27E-2] |
| I15 | Random Forest | 0 | AUPRC | 0.0031 [0.0027-0.0038] | 0.0031 [0.0028-0.0037] | [4.38E-5,7.74E-5] |
| I15 | Random Forest | 0.0015 | AUROC | 0.6294 [0.6174-0.6408] | 0.6309 [0.6198-0.6418] | [1.25E-3,2.26E-3] |
| I20 | AdaBoost | 0.3845 | AUPRC | 0.0412 [0.0252-0.05] | 0.4257 [0.2411-0.4257] | [3.77E-1,3.77E-1] |
| I20 | AdaBoost | -0.1015 | AUROC | 0.6837 [0.6812-0.6855] | 0.5822 [0.5812-0.6158] | [-1.01E-1,-1.00E-1] |
| I20 | Decision Tree | 0.4331 | AUPRC | 0.0223 [0.0196-0.0239] | 0.4554 [0.3853-0.4554] | [4.33E-1,4.33E-1] |
| I20 | Decision Tree | -0.1079 | AUROC | 0.6665 [0.6612-0.6708] | 0.5586 [0.5586-0.5773] | [-1.08E-1,-1.07E-1] |
| I20 | Neural Net | -0.0007 | AUPRC | 0.0263 [0.0247-0.0271] | 0.0256 [0.024-0.0264] | [-7.30E-4,-6.45E-4] |
| I20 | Neural Net | -0.0066 | AUROC | 0.6924 [0.6848-0.6952] | 0.6858 [0.677-0.6892] | [-6.64E-3,-6.27E-3] |
| I20 | Random Forest | -0.1506 | AUPRC | 0.192 [0.0174-0.3433] | 0.0414 [0.0191-0.1528] | [-1.49E-1,-1.44E-1] |
| I20 | Random Forest | 0.0111 | AUROC | 0.655 [0.6268-0.671] | 0.6661 [0.6592-0.6709] | [9.21E-3,1.18E-2] |
| I21 | AdaBoost | 0.0017 | AUPRC | 0.0332 [0.0324-0.0339] | 0.0349 [0.0343-0.0355] | [1.61E-3,1.68E-3] |
| I21 | AdaBoost | 0.0069 | AUROC | 0.6636 [0.6617-0.6652] | 0.6705 [0.6685-0.6722] | [6.80E-3,6.97E-3] |
| I21 | Decision Tree | -0.0003 | AUPRC | 0.0317 [0.0268-0.0341] | 0.0314 [0.0268-0.0346] | [-5.66E-5,3.16E-4] |
| I21 | Decision Tree | 0.0024 | AUROC | 0.6507 [0.6416-0.6566] | 0.6531 [0.6447-0.6594] | [2.56E-3,3.35E-3] |
| I21 | Neural Net | 0.0015 | AUPRC | 0.0413 [0.0384-0.043] | 0.0428 [0.0388-0.0446] | [1.44E-3,1.63E-3] |
| I21 | Neural Net | 0.0035 | AUROC | 0.685 [0.6807-0.6875] | 0.6885 [0.6823-0.6914] | [3.27E-3,3.59E-3] |
| I21 | Random Forest | 0.0009 | AUPRC | 0.0324 [0.0302-0.0348] | 0.0333 [0.0316-0.0353] | [7.52E-4,9.37E-4] |
| I21 | Random Forest | 0.0055 | AUROC | 0.6598 [0.6533-0.665] | 0.6653 [0.661-0.6696] | [5.51E-3,5.94E-3] |
| I24 | AdaBoost | 0.0027 | AUPRC | 0.0528 [0.0224-0.3031] | 0.0555 [0.0249-0.2912] | [0.00E+0,0.00E+0] |
| I24 | AdaBoost | 0.0038 | AUROC | 0.5803 [0.5322-0.5868] | 0.5841 [0.5402-0.5877] | [0.00E+0,0.00E+0] |
| I24 | Decision Tree | 0.0013 | AUPRC | 0.0038 [0.001-0.013] | 0.0051 [0.0011-0.0157] | [4.96E-5,1.22E-3] |
| I24 | Decision Tree | 0.0013 | AUROC | 0.5819 [0.5559-0.6013] | 0.5832 [0.5559-0.6036] | [6.95E-4,2.74E-3] |
| I24 | Neural Net | 2.00E-04 | AUPRC | 0.001 [0.0008-0.0012] | 0.0012 [0.0009-0.0014] | [2.11E-4,2.35E-4] |
| I24 | Neural Net | 0.0596 | AUROC | 0.5356 [0.4622-0.5927] | 0.5952 [0.5195-0.619] | [5.27E-2,5.81E-2] |
| I24 | Random Forest | 0 | AUPRC | 0.0013 [0.0012-0.0016] | 0.0013 [0.0012-0.0015] | [1.11E-6,8.96E-6] |
| I24 | Random Forest | 0.0027 | AUROC | 0.6165 [0.6021-0.6294] | 0.6192 [0.6059-0.6306] | [2.17E-3,3.27E-3] |
| I25 | AdaBoost | 0.0017 | AUPRC | 0.1328 [0.1328-0.1328] | 0.1345 [0.1345-0.1345] | [0.00E+0,0.00E+0] |
| I25 | AdaBoost | 0.0014 | AUROC | 0.6347 [0.6347-0.6347] | 0.6361 [0.6361-0.6361] | [0.00E+0,0.00E+0] |
| I25 | Decision Tree | -0.0032 | AUPRC | 0.0991 [0.09-0.1348] | 0.0959 [0.095-0.1343] | [-2.33E-3,-2.21E-3] |
| I25 | Decision Tree | 0.0054 | AUROC | 0.6413 [0.6246-0.6466] | 0.6467 [0.645-0.649] | [5.59E-3,6.61E-3] |
| I25 | Neural Net | 0.0002 | AUPRC | 0.075 [0.0738-0.0761] | 0.0752 [0.0737-0.0764] | [1.40E-4,2.56E-4] |
| I25 | Neural Net | 0.0024 | AUROC | 0.6883 [0.6852-0.6899] | 0.6907 [0.688-0.6924] | [2.37E-3,2.53E-3] |
| I25 | Random Forest | 0.0012 | AUPRC | 0.0697 [0.068-0.0712] | 0.0709 [0.0692-0.0724] | [1.12E-3,1.29E-3] |
| I25 | Random Forest | 0.0047 | AUROC | 0.6718 [0.6683-0.675] | 0.6765 [0.6727-0.6795] | [4.40E-3,4.76E-3] |
| I26 | AdaBoost | -0.004 | AUPRC | 0.0172 [0.0128-0.0229] | 0.0132 [0.0128-0.0137] | [-3.97E-3,-3.71E-3] |
| I26 | AdaBoost | 0.0104 | AUROC | 0.6616 [0.6577-0.6646] | 0.672 [0.6679-0.6746] | [1.02E-2,1.05E-2] |
| I26 | Decision Tree | 0.0031 | AUPRC | 0.0746 [0.0494-0.3113] | 0.0777 [0.0644-0.3338] | [0.00E+0,0.00E+0] |
| I26 | Decision Tree | 0 | AUROC | 0.6449 [0.6376-0.65] | 0.6449 [0.6379-0.6507] | [7.08E-4,1.86E-3] |
| I26 | Neural Net | 0 | AUPRC | 0.0135 [0.0122-0.0142] | 0.0135 [0.0124-0.0141] | [-9.91E-5,-2.82E-5] |
| I26 | Neural Net | 0.0018 | AUROC | 0.6759 [0.661-0.681] | 0.6777 [0.6659-0.683] | [1.47E-3,2.07E-3] |
| I26 | Random Forest | 0.0001 | AUPRC | 0.0124 [0.0117-0.013] | 0.0125 [0.0118-0.0133] | [1.07E-4,1.80E-4] |
| I26 | Random Forest | 0.0032 | AUROC | 0.6634 [0.6566-0.6685] | 0.6666 [0.6579-0.6729] | [2.97E-3,3.56E-3] |
| I27 | AdaBoost | -3.00E-04 | AUPRC | 0.0069 [0.0063-0.0075] | 0.0066 [0.0061-0.0072] | [-3.10E-4,-2.87E-4] |
| I27 | AdaBoost | 0.0007 | AUROC | 0.759 [0.7507-0.7663] | 0.7597 [0.7505-0.7676] | [5.37E-4,9.65E-4] |
| I27 | Decision Tree | 0.0125 | AUPRC | 0.0244 [0.0099-0.0482] | 0.0369 [0.0194-0.0612] | [1.07E-2,1.28E-2] |
| I27 | Decision Tree | -0.0031 | AUROC | 0.7124 [0.6865-0.7309] | 0.7093 [0.6863-0.7288] | [-3.70E-3,-1.57E-3] |
| I27 | Neural Net | -5.00E-04 | AUPRC | 0.0072 [0.0042-0.0099] | 0.0067 [0.0042-0.0091] | [-7.05E-4,-3.38E-4] |
| I27 | Neural Net | -0.0015 | AUROC | 0.7433 [0.6945-0.7643] | 0.7418 [0.6956-0.7658] | [-2.85E-3,8.63E-4] |
| I27 | Random Forest | -3.00E-04 | AUPRC | 0.007 [0.0056-0.0091] | 0.0067 [0.0057-0.0083] | [-2.64E-4,-1.05E-4] |
| I27 | Random Forest | 0.0024 | AUROC | 0.7459 [0.7341-0.7553] | 0.7483 [0.7391-0.7559] | [1.63E-3,2.45E-3] |
| I30 | AdaBoost | 0 | AUPRC | 0.0025 [0.0023-0.0028] | 0.0025 [0.0023-0.0028] | [-3.08E-5,-1.73E-5] |
| I30 | AdaBoost | -0.0031 | AUROC | 0.7001 [0.6871-0.7107] | 0.697 [0.6835-0.7087] | [-3.49E-3,-2.67E-3] |
| I30 | Decision Tree | -0.005 | AUPRC | 0.0296 [0.0169-0.0468] | 0.0246 [0.0142-0.0421] | [-5.53E-3,-3.88E-3] |
| I30 | Decision Tree | 0.004 | AUROC | 0.6449 [0.6221-0.6674] | 0.6489 [0.6272-0.6684] | [2.90E-3,4.68E-3] |
| I30 | Neural Net | 0 | AUPRC | 0.0024 [0.0019-0.0028] | 0.0024 [0.0017-0.0027] | [-6.78E-5,-2.39E-5] |
| I30 | Neural Net | -0.0032 | AUROC | 0.6909 [0.6402-0.7074] | 0.6877 [0.6193-0.7061] | [-4.61E-3,-1.69E-3] |
| I30 | Random Forest | -2.00E-04 | AUPRC | 0.0028 [0.0025-0.0032] | 0.0026 [0.0023-0.0056] | [-1.42E-4,-1.19E-4] |
| I30 | Random Forest | -0.0085 | AUROC | 0.707 [0.6969-0.7161] | 0.6985 [0.6867-0.7082] | [-8.59E-3,-7.81E-3] |
| I33 | AdaBoost | 0.0001 | AUPRC | 0.0084 [0.0077-0.0092] | 0.0085 [0.0077-0.0093] | [3.47E-5,6.00E-5] |
| I33 | AdaBoost | -0.0023 | AUROC | 0.7821 [0.7758-0.7869] | 0.7798 [0.7732-0.7853] | [-2.37E-3,-2.12E-3] |
| I33 | Decision Tree | 0.0484 | AUPRC | 0.0065 [0.0048-0.0424] | 0.0549 [0.0326-0.0807] | [4.57E-2,4.77E-2] |
| I33 | Decision Tree | -0.0291 | AUROC | 0.7536 [0.7382-0.7655] | 0.7245 [0.7048-0.7428] | [-3.03E-2,-2.88E-2] |
| I33 | Neural Net | -0.0002 | AUPRC | 0.0077 [0.0059-0.009] | 0.0075 [0.0056-0.0088] | [-2.20E-4,-6.93E-5] |
| I33 | Neural Net | -0.0025 | AUROC | 0.7831 [0.7636-0.7914] | 0.7806 [0.7565-0.7911] | [-2.79E-3,-1.78E-3] |
| I33 | Random Forest | -0.0002 | AUPRC | 0.0074 [0.0068-0.0082] | 0.0072 [0.0066-0.0083] | [-2.27E-4,-1.63E-4] |
| I33 | Random Forest | -0.0019 | AUROC | 0.7795 [0.7747-0.7839] | 0.7776 [0.7718-0.7829] | [-2.27E-3,-1.85E-3] |
| I34 | AdaBoost | -2.00E-04 | AUPRC | 0.0053 [0.005-0.0056] | 0.0051 [0.0049-0.0054] | [-1.73E-4,-1.62E-4] |
| I34 | AdaBoost | -0.0047 | AUROC | 0.7062 [0.6992-0.7123] | 0.7015 [0.6948-0.7075] | [-4.83E-3,-4.46E-3] |
| I34 | Decision Tree | -0.0002 | AUPRC | 0.0097 [0.0055-0.0199] | 0.0095 [0.0053-0.0189] | [-3.47E-4,2.93E-4] |
| I34 | Decision Tree | -0.0045 | AUROC | 0.6561 [0.6418-0.6693] | 0.6516 [0.6326-0.6676] | [-5.08E-3,-3.60E-3] |
| I34 | Neural Net | -1.00E-04 | AUPRC | 0.0052 [0.0037-0.006] | 0.0051 [0.0036-0.0059] | [-1.37E-4,-3.73E-5] |
| I34 | Neural Net | -0.0006 | AUROC | 0.6898 [0.648-0.7028] | 0.6892 [0.6433-0.7018] | [-1.86E-3,3.40E-5] |
| I34 | Random Forest | 1.00E-04 | AUPRC | 0.0046 [0.0041-0.0063] | 0.0047 [0.0043-0.0059] | [5.07E-5,1.06E-4] |
| I34 | Random Forest | 0.0025 | AUROC | 0.671 [0.6527-0.6858] | 0.6735 [0.6601-0.6859] | [2.15E-3,3.69E-3] |
| I35 | AdaBoost | 0.0002 | AUPRC | 0.0217 [0.0212-0.0221] | 0.0219 [0.0214-0.0222] | [1.65E-4,1.94E-4] |
| I35 | AdaBoost | 0.0045 | AUROC | 0.715 [0.7121-0.7171] | 0.7195 [0.7167-0.7215] | [4.41E-3,4.59E-3] |
| I35 | Decision Tree | 0.0003 | AUPRC | 0.0242 [0.0206-0.0285] | 0.0245 [0.0211-0.0292] | [2.09E-4,5.92E-4] |
| I35 | Decision Tree | 0.0055 | AUROC | 0.6768 [0.6697-0.6846] | 0.6823 [0.6742-0.6894] | [4.90E-3,5.82E-3] |
| I35 | Neural Net | 0.0008 | AUPRC | 0.0236 [0.0214-0.0245] | 0.0244 [0.0225-0.0254] | [6.80E-4,7.59E-4] |
| I35 | Neural Net | 0.0069 | AUROC | 0.7261 [0.7122-0.7298] | 0.733 [0.7219-0.7365] | [6.67E-3,7.05E-3] |
| I35 | Random Forest | -0.0011 | AUPRC | 0.0194 [0.0185-0.0205] | 0.0183 [0.0171-0.0212] | [-1.14E-3,-1.04E-3] |
| I35 | Random Forest | -0.0124 | AUROC | 0.6909 [0.6833-0.6981] | 0.6785 [0.6614-0.6908] | [-1.29E-2,-1.16E-2] |
| I42 | AdaBoost | -1.00E-04 | AUPRC | 0.007 [0.0065-0.0075] | 0.0069 [0.0064-0.0075] | [-9.04E-5,-5.50E-5] |
| I42 | AdaBoost | -0.001 | AUROC | 0.671 [0.6636-0.676] | 0.67 [0.6611-0.6762] | [-1.09E-3,-6.39E-4] |
| I42 | Decision Tree | 0.0154 | AUPRC | 0.0128 [0.0071-0.0258] | 0.0282 [0.0166-0.0427] | [1.37E-2,1.48E-2] |
| I42 | Decision Tree | -0.002 | AUROC | 0.6386 [0.6256-0.6501] | 0.6366 [0.6216-0.6498] | [-2.62E-3,-1.35E-3] |
| I42 | Neural Net | 0.0004 | AUPRC | 0.0064 [0.0049-0.0075] | 0.0068 [0.005-0.008] | [2.97E-4,4.20E-4] |
| I42 | Neural Net | 0.0065 | AUROC | 0.6735 [0.6478-0.6835] | 0.68 [0.6556-0.6885] | [5.73E-3,6.75E-3] |
| I42 | Random Forest | 0.0074 | AUPRC | 0.0063 [0.0056-0.0073] | 0.0137 [0.0049-0.1092] | [5.42E-3,9.12E-3] |
| I42 | Random Forest | -0.0473 | AUROC | 0.6657 [0.6593-0.6712] | 0.6184 [0.5819-0.6437] | [-4.88E-2,-4.46E-2] |
| I44 | AdaBoost | 0.0002 | AUPRC | 0.0089 [0.0086-0.0109] | 0.0091 [0.0086-0.0115] | [1.45E-4,2.03E-4] |
| I44 | AdaBoost | 0.0024 | AUROC | 0.6906 [0.685-0.695] | 0.693 [0.6873-0.6976] | [2.08E-3,2.42E-3] |
| I44 | Decision Tree | 0.0009 | AUPRC | 0.0073 [0.0062-0.0185] | 0.0082 [0.0066-0.0115] | [6.71E-4,8.63E-4] |
| I44 | Decision Tree | 0.0124 | AUROC | 0.6469 [0.634-0.6555] | 0.6593 [0.6482-0.6672] | [1.18E-2,1.28E-2] |
| I44 | Neural Net | 3.00E-04 | AUPRC | 0.0091 [0.0082-0.0098] | 0.0094 [0.0082-0.0102] | [2.98E-4,3.67E-4] |
| I44 | Neural Net | 0.0087 | AUROC | 0.6921 [0.6775-0.6985] | 0.7008 [0.686-0.7067] | [8.21E-3,8.94E-3] |
| I44 | Random Forest | 0.0001 | AUPRC | 0.0087 [0.0082-0.0097] | 0.0088 [0.0084-0.0099] | [9.14E-5,1.37E-4] |
| I44 | Random Forest | 0.0066 | AUROC | 0.6866 [0.6799-0.6922] | 0.6932 [0.6875-0.6982] | [6.28E-3,6.75E-3] |
| I45 | AdaBoost | -0.3731 | AUPRC | 0.3754 [0.3751-0.4672] | 0.0023 [0.0021-0.0025] | [-3.73E-1,-3.73E-1] |
| I45 | AdaBoost | 0.0665 | AUROC | 0.5581 [0.5295-0.5581] | 0.6246 [0.6106-0.6372] | [6.75E-2,6.88E-2] |
| I45 | Decision Tree | -0.3564 | AUPRC | 0.3754 [0.3754-0.4672] | 0.019 [0.01-0.0313] | [-3.57E-1,-3.56E-1] |
| I45 | Decision Tree | 0.0327 | AUROC | 0.5581 [0.5295-0.5581] | 0.5908 [0.5679-0.6128] | [3.34E-2,3.52E-2] |
| I45 | Neural Net | 0 | AUPRC | 0.0021 [0.0014-0.0025] | 0.0021 [0.0015-0.0024] | [-4.76E-5,6.61E-6] |
| I45 | Neural Net | -0.0053 | AUROC | 0.613 [0.5155-0.6379] | 0.6077 [0.536-0.6297] | [-6.17E-3,-2.51E-3] |
| I45 | Random Forest | 0.4575 | AUPRC | 0.0022 [0.0011-0.0669] | 0.4597 [0.0131-0.4933] | [4.13E-1,4.60E-1] |
| I45 | Random Forest | -0.0507 | AUROC | 0.5755 [0.5376-0.6065] | 0.5248 [0.5025-0.553] | [-4.82E-2,-4.44E-2] |
| I46 | AdaBoost | 0.001 | AUPRC | 0.038 [0.0337-0.0422] | 0.039 [0.0345-0.044] | [9.78E-4,1.21E-3] |
| I46 | AdaBoost | -0.0004 | AUROC | 0.8174 [0.8143-0.8202] | 0.817 [0.8128-0.8203] | [-4.20E-4,-1.87E-4] |
| I46 | Decision Tree | 0.0519 | AUPRC | 0.0262 [0.0193-0.0667] | 0.0781 [0.041-0.1064] | [4.44E-2,4.78E-2] |
| I46 | Decision Tree | -0.0053 | AUROC | 0.7829 [0.7723-0.7923] | 0.7776 [0.7649-0.7888] | [-5.69E-3,-4.73E-3] |
| I46 | Neural Net | 0.0002 | AUPRC | 0.0441 [0.0267-0.0582] | 0.0443 [0.0261-0.0584] | [-5.92E-5,1.27E-3] |
| I46 | Neural Net | -1.00E-04 | AUROC | 0.8152 [0.7985-0.8225] | 0.8151 [0.7975-0.8227] | [-2.83E-4,6.86E-4] |
| I46 | Random Forest | -0.0008 | AUPRC | 0.0342 [0.0291-0.0428] | 0.0334 [0.0285-0.0414] | [-1.04E-3,-5.02E-4] |
| I46 | Random Forest | -0.0009 | AUROC | 0.8099 [0.8053-0.8138] | 0.809 [0.8049-0.8129] | [-1.00E-3,-6.44E-4] |
| I47 | AdaBoost | -0.01 | AUPRC | 0.3974 [0.3758-0.46] | 0.3874 [0.3874-0.4573] | [0.00E+0,0.00E+0] |
| I47 | AdaBoost | 0.0013 | AUROC | 0.5596 [0.5313-0.5596] | 0.5609 [0.5314-0.5609] | [0.00E+0,0.00E+0] |
| I47 | Decision Tree | -0.01 | AUPRC | 0.3974 [0.3758-0.46] | 0.3874 [0.3874-0.4573] | [0.00E+0,0.00E+0] |
| I47 | Decision Tree | 0.0013 | AUROC | 0.5596 [0.5313-0.5596] | 0.5609 [0.5314-0.5609] | [0.00E+0,0.00E+0] |
| I47 | Neural Net | 0 | AUPRC | 0.0098 [0.0091-0.0103] | 0.0098 [0.0093-0.0102] | [-3.50E-5,8.48E-6] |
| I47 | Neural Net | -0.0004 | AUROC | 0.6309 [0.6171-0.6372] | 0.6305 [0.6201-0.6365] | [-6.58E-4,-1.05E-5] |
| I47 | Random Forest | -0.0915 | AUPRC | 0.46 [0.0256-0.496] | 0.3685 [0.0208-0.4715] | [-7.26E-2,-5.29E-2] |
| I47 | Random Forest | 0.0202 | AUROC | 0.5294 [0.5053-0.5596] | 0.5496 [0.5187-0.5821] | [0.00E+0,0.00E+0] |
| I48 | AdaBoost | -0.0013 | AUPRC | 0.1703 [0.1691-0.1715] | 0.169 [0.1672-0.1703] | [-1.50E-3,-1.36E-3] |
| I48 | AdaBoost | 0.0033 | AUROC | 0.7057 [0.7051-0.7064] | 0.709 [0.7083-0.7095] | [3.18E-3,3.23E-3] |
| I48 | Decision Tree | -0.0058 | AUPRC | 0.1589 [0.1564-0.1612] | 0.1531 [0.1507-0.1556] | [-5.87E-3,-5.65E-3] |
| I48 | Decision Tree | 0.0004 | AUROC | 0.6971 [0.6949-0.6991] | 0.6975 [0.6951-0.6997] | [1.61E-4,3.82E-4] |
| I48 | Neural Net | -0.0016 | AUPRC | 0.1854 [0.1821-0.1873] | 0.1838 [0.181-0.1858] | [-1.69E-3,-1.46E-3] |
| I48 | Neural Net | 0.0035 | AUROC | 0.7184 [0.7161-0.7197] | 0.7219 [0.7202-0.7232] | [3.44E-3,3.60E-3] |
| I48 | Random Forest | -0.0023 | AUPRC | 0.1602 [0.1472-0.1667] | 0.1579 [0.1414-0.1659] | [-2.64E-3,-1.66E-3] |
| I48 | Random Forest | 0.0021 | AUROC | 0.6858 [0.6754-0.6929] | 0.6879 [0.6772-0.6956] | [1.36E-3,2.33E-3] |
| I49 | AdaBoost | 0.4153 | AUPRC | 0.0117 [0.0112-0.0121] | 0.427 [0.4162-0.4319] | [4.15E-1,4.15E-1] |
| I49 | AdaBoost | -0.0878 | AUROC | 0.6649 [0.6607-0.6685] | 0.5771 [0.5702-0.5771] | [-8.81E-2,-8.77E-2] |
| I49 | Decision Tree | -0.0013 | AUPRC | 0.0093 [0.0073-0.0111] | 0.008 [0.005-0.3974] | [-1.06E-3,-7.71E-4] |
| I49 | Decision Tree | -0.0173 | AUROC | 0.6243 [0.6124-0.6323] | 0.607 [0.5998-0.615] | [-1.67E-2,-1.58E-2] |
| I49 | Neural Net | 1.00E-04 | AUPRC | 0.0122 [0.0114-0.0128] | 0.0123 [0.0115-0.0129] | [5.43E-5,9.70E-5] |
| I49 | Neural Net | 0.0043 | AUROC | 0.6736 [0.664-0.6789] | 0.6779 [0.6676-0.6826] | [3.99E-3,4.51E-3] |
| I49 | Random Forest | -0.3661 | AUPRC | 0.3739 [0.0805-0.4843] | 0.0078 [0.0046-0.3875] | [-3.63E-1,-3.48E-1] |
| I49 | Random Forest | 0.0351 | AUROC | 0.5603 [0.5249-0.581] | 0.5954 [0.5663-0.6116] | [3.24E-2,3.59E-2] |
| I50 | AdaBoost | 0.0018 | AUPRC | 0.1569 [0.1569-0.1569] | 0.1587 [0.1587-0.1587] | [0.00E+0,0.00E+0] |
| I50 | AdaBoost | 0.0016 | AUROC | 0.6658 [0.6658-0.6658] | 0.6674 [0.6674-0.6674] | [0.00E+0,0.00E+0] |
| I50 | Decision Tree | -0.0002 | AUPRC | 0.086 [0.083-0.0893] | 0.0858 [0.0828-0.0891] | [-3.27E-4,-1.52E-5] |
| I50 | Decision Tree | 0.0026 | AUROC | 0.7293 [0.7256-0.7325] | 0.7319 [0.7282-0.735] | [2.42E-3,2.81E-3] |
| I50 | Neural Net | 0.0008 | AUPRC | 0.0965 [0.0943-0.0981] | 0.0973 [0.0951-0.0991] | [7.20E-4,9.05E-4] |
| I50 | Neural Net | 0.0038 | AUROC | 0.7519 [0.7497-0.7534] | 0.7557 [0.7537-0.757] | [3.72E-3,3.85E-3] |
| I50 | Random Forest | 0.0033 | AUPRC | 0.0771 [0.0732-0.0812] | 0.0804 [0.0771-0.0837] | [3.05E-3,3.52E-3] |
| I50 | Random Forest | 0.0084 | AUROC | 0.7236 [0.7158-0.7297] | 0.732 [0.7266-0.7369] | [8.16E-3,8.83E-3] |
| I51 | AdaBoost | 1.00E-04 | AUPRC | 0.0024 [0.0021-0.0034] | 0.0025 [0.0022-0.0027] | [2.39E-5,4.10E-5] |
| I51 | AdaBoost | 0.0127 | AUROC | 0.6243 [0.6052-0.6403] | 0.637 [0.622-0.651] | [1.21E-2,1.29E-2] |
| I51 | Decision Tree | 0.0016 | AUPRC | 0.0288 [0.0155-0.0471] | 0.0304 [0.0161-0.0479] | [7.94E-4,2.40E-3] |
| I51 | Decision Tree | 0.0013 | AUROC | 0.5907 [0.5625-0.6133] | 0.592 [0.5659-0.6149] | [4.47E-4,2.49E-3] |
| I51 | Neural Net | 0 | AUPRC | 0.0023 [0.0018-0.0026] | 0.0023 [0.0018-0.0026] | [-3.94E-6,3.35E-5] |
| I51 | Neural Net | 0.0034 | AUROC | 0.6203 [0.5659-0.6401] | 0.6237 [0.5599-0.6426] | [1.47E-3,4.59E-3] |
| I51 | Random Forest | -1.00E-04 | AUPRC | 0.0024 [0.0022-0.0028] | 0.0023 [0.0021-0.0027] | [-1.02E-4,-7.93E-5] |
| I51 | Random Forest | -0.0052 | AUROC | 0.6335 [0.618-0.6464] | 0.6283 [0.6074-0.6444] | [-6.48E-3,-4.81E-3] |
| I60 | AdaBoost | -3.00E-04 | AUPRC | 0.0072 [0.0059-0.0107] | 0.0069 [0.0056-0.0107] | [-4.20E-4,-3.31E-4] |
| I60 | AdaBoost | -0.0013 | AUROC | 0.7067 [0.6976-0.7152] | 0.7054 [0.6949-0.7137] | [-1.72E-3,-1.13E-3] |
| I60 | Decision Tree | -0.0388 | AUPRC | 0.0758 [0.0508-0.1049] | 0.037 [0.0165-0.0662] | [-3.89E-2,-3.67E-2] |
| I60 | Decision Tree | 0.0077 | AUROC | 0.6508 [0.6263-0.6733] | 0.6585 [0.6336-0.6796] | [6.68E-3,8.59E-3] |
| I60 | Neural Net | 0.0004 | AUPRC | 0.0075 [0.0043-0.011] | 0.0079 [0.0043-0.0119] | [2.73E-4,5.84E-4] |
| I60 | Neural Net | -0.0025 | AUROC | 0.7049 [0.6691-0.7171] | 0.7024 [0.6661-0.7143] | [-3.39E-3,-1.88E-3] |
| I60 | Random Forest | 1.00E-04 | AUPRC | 0.0073 [0.0061-0.0101] | 0.0074 [0.006-0.0102] | [-5.78E-5,9.87E-5] |
| I60 | Random Forest | -0.001 | AUROC | 0.7041 [0.6944-0.7132] | 0.7031 [0.6939-0.7111] | [-1.59E-3,-7.72E-4] |
| I61 | AdaBoost | -4.00E-04 | AUPRC | 0.007 [0.0065-0.0076] | 0.0066 [0.0062-0.007] | [-3.91E-4,-3.63E-4] |
| I61 | AdaBoost | 0.0033 | AUROC | 0.6469 [0.64-0.6526] | 0.6502 [0.644-0.6561] | [3.15E-3,3.53E-3] |
| I61 | Decision Tree | -0.0214 | AUPRC | 0.0657 [0.0512-0.08] | 0.0443 [0.0314-0.0577] | [-2.20E-2,-2.07E-2] |
| I61 | Decision Tree | 0.0078 | AUROC | 0.5973 [0.5801-0.6138] | 0.6051 [0.5891-0.6206] | [7.27E-3,8.94E-3] |
| I61 | Neural Net | -2.00E-04 | AUPRC | 0.0076 [0.0061-0.0087] | 0.0074 [0.006-0.0085] | [-2.35E-4,-1.22E-4] |
| I61 | Neural Net | 0.005 | AUROC | 0.6578 [0.6322-0.6687] | 0.6628 [0.6392-0.6724] | [4.19E-3,5.03E-3] |
| I61 | Random Forest | -1.00E-04 | AUPRC | 0.0061 [0.0053-0.0083] | 0.006 [0.0052-0.0079] | [-2.12E-4,-1.32E-4] |
| I61 | Random Forest | 0.0033 | AUROC | 0.6227 [0.6081-0.6362] | 0.626 [0.6105-0.6386] | [2.02E-3,3.58E-3] |
| I63 | AdaBoost | 0.0845 | AUPRC | 0.3958 [0.3958-0.4814] | 0.4803 [0.384-0.4803] | [0.00E+0,0.00E+0] |
| I63 | AdaBoost | -0.0261 | AUROC | 0.5516 [0.5245-0.5516] | 0.5255 [0.5255-0.5509] | [0.00E+0,0.00E+0] |
| I63 | Decision Tree | -0.0011 | AUPRC | 0.4814 [0.3958-0.5052] | 0.4803 [0.384-0.4803] | [0.00E+0,0.00E+0] |
| I63 | Decision Tree | 0.001 | AUROC | 0.5245 [0.5065-0.5516] | 0.5255 [0.5255-0.5509] | [0.00E+0,0.00E+0] |
| I63 | Neural Net | 0.0002 | AUPRC | 0.0292 [0.0282-0.0299] | 0.0294 [0.0286-0.0302] | [1.63E-4,2.15E-4] |
| I63 | Neural Net | 0.0045 | AUROC | 0.6531 [0.6467-0.6562] | 0.6576 [0.6534-0.6604] | [4.25E-3,4.53E-3] |
| I63 | Random Forest | -0.4159 | AUPRC | 0.4416 [0.0408-0.4981] | 0.0257 [0.0249-0.0265] | [-4.18E-1,-3.99E-1] |
| I63 | Random Forest | 0.0901 | AUROC | 0.5331 [0.5136-0.5668] | 0.6232 [0.6185-0.6279] | [8.80E-2,9.01E-2] |
| I64 | AdaBoost | 0.0004 | AUPRC | 0.0118 [0.0115-0.0121] | 0.0122 [0.0119-0.0126] | [3.77E-4,3.94E-4] |
| I64 | AdaBoost | 0.0105 | AUROC | 0.6142 [0.6092-0.6184] | 0.6247 [0.6197-0.6285] | [1.05E-2,1.07E-2] |
| I64 | Decision Tree | 0.0153 | AUPRC | 0.0117 [0.0106-0.0137] | 0.027 [0.0219-0.0334] | [1.49E-2,1.54E-2] |
| I64 | Decision Tree | 0.0046 | AUROC | 0.5977 [0.5894-0.6047] | 0.6023 [0.5936-0.6107] | [4.22E-3,5.02E-3] |
| I64 | Neural Net | 6.00E-04 | AUPRC | 0.0119 [0.011-0.0125] | 0.0125 [0.0116-0.0131] | [5.07E-4,5.74E-4] |
| I64 | Neural Net | 0.0142 | AUROC | 0.6171 [0.6034-0.6241] | 0.6313 [0.6201-0.6368] | [1.35E-2,1.42E-2] |
| I64 | Random Forest | 3.00E-04 | AUPRC | 0.0118 [0.0113-0.0122] | 0.0121 [0.0117-0.0126] | [3.70E-4,4.03E-4] |
| I64 | Random Forest | 0.012 | AUROC | 0.613 [0.609-0.6171] | 0.625 [0.6214-0.6283] | [1.17E-2,1.20E-2] |
| I69 | AdaBoost | 1.00E-04 | AUPRC | 0.0304 [0.03-0.0308] | 0.0305 [0.0301-0.0309] | [1.18E-4,1.43E-4] |
| I69 | AdaBoost | 0.0041 | AUROC | 0.625 [0.6224-0.6273] | 0.6291 [0.6271-0.6313] | [4.10E-3,4.25E-3] |
| I69 | Decision Tree | 0.0106 | AUPRC | 0.0286 [0.0253-0.0337] | 0.0392 [0.0365-0.042] | [1.04E-2,1.07E-2] |
| I69 | Decision Tree | 0.0133 | AUROC | 0.6039 [0.5928-0.6087] | 0.6172 [0.6117-0.6222] | [1.33E-2,1.40E-2] |
| I69 | Neural Net | 0.0003 | AUPRC | 0.0321 [0.0313-0.0327] | 0.0324 [0.0317-0.033] | [2.67E-4,3.19E-4] |
| I69 | Neural Net | 0.0041 | AUROC | 0.6401 [0.6363-0.643] | 0.6442 [0.6402-0.6472] | [4.04E-3,4.28E-3] |
| I69 | Random Forest | 0.0002 | AUPRC | 0.0297 [0.029-0.0304] | 0.0299 [0.0291-0.0307] | [1.71E-4,2.29E-4] |
| I69 | Random Forest | 0.0025 | AUROC | 0.6236 [0.6209-0.6261] | 0.6261 [0.6226-0.6294] | [2.47E-3,2.74E-3] |
| I70 | AdaBoost | 0.0001 | AUPRC | 0.0169 [0.0164-0.0174] | 0.017 [0.0165-0.0176] | [8.64E-5,1.11E-4] |
| I70 | AdaBoost | 0.0006 | AUROC | 0.7257 [0.7232-0.7279] | 0.7263 [0.7238-0.7287] | [5.00E-4,6.33E-4] |
| I70 | Decision Tree | 0.0021 | AUPRC | 0.0112 [0.0092-0.0456] | 0.0133 [0.0115-0.016] | [2.05E-3,2.21E-3] |
| I70 | Decision Tree | 0.0096 | AUROC | 0.6842 [0.6737-0.6917] | 0.6938 [0.6844-0.703] | [9.60E-3,1.07E-2] |
| I70 | Neural Net | 5.00E-04 | AUPRC | 0.017 [0.0159-0.0179] | 0.0175 [0.0161-0.0185] | [3.85E-4,4.67E-4] |
| I70 | Neural Net | 0.0039 | AUROC | 0.7317 [0.7197-0.7361] | 0.7356 [0.7231-0.74] | [3.63E-3,4.09E-3] |
| I70 | Random Forest | 8.00E-04 | AUPRC | 0.0151 [0.0139-0.0174] | 0.0159 [0.0149-0.0177] | [7.67E-4,9.09E-4] |
| I70 | Random Forest | 0.0057 | AUROC | 0.7099 [0.703-0.7161] | 0.7156 [0.7103-0.7204] | [5.43E-3,5.99E-3] |
| I71 | AdaBoost | 0.0027 | AUPRC | 0.102 [0.1009-0.102] | 0.1047 [0.1036-0.1047] | [0.00E+0,0.00E+0] |
| I71 | AdaBoost | 0.0011 | AUROC | 0.6541 [0.632-0.6541] | 0.6552 [0.6332-0.6552] | [0.00E+0,0.00E+0] |
| I71 | Decision Tree | 0.0076 | AUPRC | 0.0157 [0.0068-0.0939] | 0.0233 [0.0141-0.0339] | [5.99E-3,8.44E-3] |
| I71 | Decision Tree | 0.0048 | AUROC | 0.6685 [0.6567-0.6797] | 0.6733 [0.661-0.6846] | [3.96E-3,5.27E-3] |
| I71 | Neural Net | 0 | AUPRC | 0.0116 [0.0093-0.0142] | 0.0116 [0.0089-0.0138] | [-1.65E-4,-3.81E-6] |
| I71 | Neural Net | 0.003 | AUROC | 0.7115 [0.6904-0.7185] | 0.7145 [0.6856-0.7215] | [2.16E-3,3.06E-3] |
| I71 | Random Forest | 0.0002 | AUPRC | 0.0115 [0.0089-0.0158] | 0.0117 [0.0092-0.0162] | [9.73E-5,4.29E-4] |
| I71 | Random Forest | 0.008 | AUROC | 0.6829 [0.6709-0.6923] | 0.6909 [0.6797-0.6998] | [7.61E-3,8.40E-3] |
| I73 | AdaBoost | -0.0019 | AUPRC | 0.4463 [0.1195-0.4463] | 0.4444 [0.1211-0.4444] | [0.00E+0,0.00E+0] |
| I73 | AdaBoost | 0.0031 | AUROC | 0.5869 [0.5869-0.5979] | 0.59 [0.59-0.6003] | [0.00E+0,0.00E+0] |
| I73 | Decision Tree | 0.0016 | AUPRC | 0.1169 [0.0036-0.4385] | 0.1185 [0.0037-0.4363] | [1.61E-3,1.62E-3] |
| I73 | Decision Tree | 0.0026 | AUROC | 0.6182 [0.5996-0.62] | 0.6208 [0.6029-0.6228] | [2.91E-3,3.03E-3] |
| I73 | Neural Net | 0.001 | AUPRC | 0.0102 [0.0081-0.0111] | 0.0112 [0.009-0.0123] | [1.01E-3,1.11E-3] |
| I73 | Neural Net | 0.0114 | AUROC | 0.7131 [0.6804-0.7213] | 0.7245 [0.7012-0.7318] | [1.04E-2,1.16E-2] |
| I73 | Random Forest | 8.00E-04 | AUPRC | 0.0087 [0.0074-0.0361] | 0.0095 [0.0082-0.034] | [6.80E-4,8.24E-4] |
| I73 | Random Forest | 0.0098 | AUROC | 0.6815 [0.6656-0.6917] | 0.6913 [0.6807-0.6997] | [9.63E-3,1.06E-2] |
| I74 | AdaBoost | 0.0266 | AUPRC | 0.0117 [0.0016-0.0943] | 0.0383 [0.0016-0.0943] | [0.00E+0,0.00E+0] |
| I74 | AdaBoost | 0.0055 | AUROC | 0.6386 [0.6207-0.6568] | 0.6441 [0.6214-0.6624] | [4.70E-3,5.64E-3] |
| I74 | Decision Tree | -0.0025 | AUPRC | 0.0141 [0.0039-0.0294] | 0.0116 [0.0013-0.1908] | [-3.66E-3,-1.29E-3] |
| I74 | Decision Tree | 0.0132 | AUROC | 0.6219 [0.59-0.6488] | 0.6351 [0.605-0.656] | [1.13E-2,1.40E-2] |
| I74 | Neural Net | 0 | AUPRC | 0.0016 [0.0011-0.002] | 0.0016 [0.0011-0.002] | [-5.78E-6,3.58E-5] |
| I74 | Neural Net | 0.0022 | AUROC | 0.6374 [0.542-0.6655] | 0.6396 [0.5523-0.6702] | [1.15E-3,5.43E-3] |
| I74 | Random Forest | 1.00E-04 | AUPRC | 0.0017 [0.0015-0.0019] | 0.0018 [0.0016-0.0021] | [5.30E-5,7.20E-5] |
| I74 | Random Forest | 0.0106 | AUROC | 0.6484 [0.6309-0.6609] | 0.659 [0.6421-0.6736] | [1.07E-2,1.20E-2] |
| I80 | AdaBoost | -5.00E-04 | AUPRC | 0.0241 [0.0195-0.0293] | 0.0236 [0.0138-0.0299] | [-3.88E-4,-2.53E-4] |
| I80 | AdaBoost | 0.0019 | AUROC | 0.6051 [0.5971-0.6099] | 0.607 [0.5998-0.6117] | [1.88E-3,2.25E-3] |
| I80 | Decision Tree | -0.0014 | AUPRC | 0.0158 [0.0138-0.0181] | 0.0144 [0.0129-0.0165] | [-1.44E-3,-1.20E-3] |
| I80 | Decision Tree | 0.002 | AUROC | 0.5975 [0.5891-0.6045] | 0.5995 [0.591-0.6064] | [1.75E-3,2.47E-3] |
| I80 | Neural Net | -0.0002 | AUPRC | 0.0149 [0.0136-0.0156] | 0.0147 [0.0133-0.0154] | [-2.96E-4,-2.22E-4] |
| I80 | Neural Net | 0.0012 | AUROC | 0.6238 [0.614-0.6293] | 0.625 [0.613-0.6311] | [9.57E-4,1.57E-3] |
| I80 | Random Forest | 0.4371 | AUPRC | 0.0277 [0.0189-0.1539] | 0.4648 [0.0788-0.4975] | [3.80E-1,3.95E-1] |
| I80 | Random Forest | -0.0804 | AUROC | 0.596 [0.5731-0.6075] | 0.5156 [0.5042-0.547] | [-7.88E-2,-7.68E-2] |
| I82 | AdaBoost | 0.0025 | AUPRC | 0.0636 [0.0636-0.0643] | 0.0661 [0.0661-0.1027] | [0.00E+0,0.00E+0] |
| I82 | AdaBoost | -0.0006 | AUROC | 0.593 [0.5554-0.593] | 0.5924 [0.5388-0.5924] | [0.00E+0,0.00E+0] |
| I82 | Decision Tree | -0.046 | AUPRC | 0.0636 [0.0103-0.237] | 0.0176 [0.0024-0.1024] | [-3.96E-2,-3.34E-2] |
| I82 | Decision Tree | 0.0026 | AUROC | 0.5867 [0.5515-0.593] | 0.5893 [0.5528-0.6023] | [2.60E-3,4.39E-3] |
| I82 | Neural Net | 3.00E-04 | AUPRC | 0.0029 [0.0019-0.0035] | 0.0032 [0.0025-0.0037] | [2.77E-4,3.38E-4] |
| I82 | Neural Net | 0.0146 | AUROC | 0.6039 [0.5109-0.6284] | 0.6185 [0.5772-0.6355] | [1.23E-2,1.54E-2] |
| I82 | Random Forest | 1.00E-04 | AUPRC | 0.0032 [0.0029-0.0036] | 0.0033 [0.003-0.0037] | [8.65E-5,1.13E-4] |
| I82 | Random Forest | 0.0054 | AUROC | 0.614 [0.6006-0.6272] | 0.6194 [0.607-0.6307] | [4.71E-3,5.61E-3] |
| I85 | AdaBoost | -0.0018 | AUPRC | 0.0501 [0.0446-0.0561] | 0.0483 [0.0429-0.0542] | [-1.98E-3,-1.76E-3] |
| I85 | AdaBoost | 0.0002 | AUROC | 0.9117 [0.9059-0.9165] | 0.9119 [0.9056-0.916] | [-2.72E-4,7.23E-5] |
| I85 | Decision Tree | -0.1157 | AUPRC | 0.2166 [0.1445-0.2731] | 0.1009 [0.0353-0.1758] | [-1.14E-1,-1.09E-1] |
| I85 | Decision Tree | 0.0272 | AUROC | 0.8567 [0.8354-0.8746] | 0.8839 [0.8706-0.8938] | [2.59E-2,2.73E-2] |
| I85 | Neural Net | -0.0007 | AUPRC | 0.0413 [0.0241-0.055] | 0.0406 [0.0234-0.0538] | [-2.15E-3,-4.73E-4] |
| I85 | Neural Net | -0.0006 | AUROC | 0.9065 [0.884-0.9143] | 0.9059 [0.8844-0.9134] | [-1.70E-3,-3.40E-4] |
| I85 | Random Forest | 0.0013 | AUPRC | 0.04 [0.0337-0.0469] | 0.0413 [0.0374-0.0457] | [1.10E-3,1.63E-3] |
| I85 | Random Forest | 0.0019 | AUROC | 0.9061 [0.9002-0.9106] | 0.908 [0.9045-0.911] | [1.70E-3,2.20E-3] |
| I95 | AdaBoost | 0 | AUPRC | 0.0073 [0.0071-0.0076] | 0.0073 [0.0071-0.0075] | [-4.23E-5,-3.40E-5] |
| I95 | AdaBoost | 0.0047 | AUROC | 0.6797 [0.6744-0.6838] | 0.6844 [0.6796-0.6885] | [4.61E-3,4.86E-3] |
| I95 | Decision Tree | -0.0026 | AUPRC | 0.0108 [0.0067-0.0419] | 0.0082 [0.0066-0.0244] | [-2.43E-3,-1.11E-3] |
| I95 | Decision Tree | 0.017 | AUROC | 0.6498 [0.6333-0.6544] | 0.6668 [0.6522-0.6731] | [1.69E-2,1.75E-2] |
| I95 | Neural Net | 0 | AUPRC | 0.0075 [0.0067-0.0081] | 0.0075 [0.0067-0.0081] | [-1.71E-5,4.34E-5] |
| I95 | Neural Net | 0.0046 | AUROC | 0.6801 [0.6618-0.688] | 0.6847 [0.667-0.6923] | [3.82E-3,5.04E-3] |
| I95 | Random Forest | 1.00E-04 | AUPRC | 0.0074 [0.007-0.008] | 0.0075 [0.0071-0.0079] | [2.99E-5,6.35E-5] |
| I95 | Random Forest | 0.0046 | AUROC | 0.6822 [0.6767-0.6865] | 0.6868 [0.6823-0.6907] | [4.45E-3,4.76E-3] |

**Supplementary Table 5 (a+b):** Feature importance by mean absolute SHAP value for versions 1 and 2 Neural Net model. Feature Importance reported across 24 features and 35 level 3 ICD-10 codes.

Table 5b- Version 2 absolute mean SHAP contribution

Table 5a- Version 1 absolute mean SHAP contribution

**Supplementary Figure 1:** compliment figure to figure 3a showing the median AUROC performance metric values for the version 1 neural net model. Dots indicate the median AUROC values and lines show the corresponding 95% CIs. The symbol (*) indicates that version 1 performed better than version 2 with respect to the calculated accelerated bootstrap gains 95% CIs and the symbol indicates the two models performed the same statistically and the symbol (•) indicates that the models performed the same statistically; no symbol indicates the model performed significantly worse (with respect to accelerated bootstrap gains 95% CIs).
